# A Batch-Processing Pipeline for RNA-seq Gene Abundance Estimation

**DOI:** 10.21769/BioProtoc.5857

**Published:** 2026-11-05

**Authors:** Juan S. Zambrano Leiton, Karen E. López, Angie F. Riascos-España, Claudia E. Salazar Gonzalez, Carlos A. Betancourth García, Pedro A. Velasquez-Vasconez

**Affiliations:** 1 Department of Production and Plant Protection, University of Nariño, Pasto, Colombia; 2 Faculty of Exact and Natural Sciences, Biology Program, University of Nariño, Pasto, Colombia; 3 School of Basic Sciences, Technology and Engineering, National Open and Distance University, Pasto, Colombia

**Keywords:** Transcriptomics, Gene expression quantification, Transcripts per million (TPM), Sequence read archive, STAR, RSEM, Data mining

## Abstract

RNA sequencing (RNA-seq) datasets provide valuable opportunities to investigate gene and transcript abundance across species, tissues, developmental stages, and experimental conditions. However, processing multiple datasets consistently remains challenging because sequencing runs may differ in library layout, read length, sequencing depth, metadata quality, and analytical settings. Existing RNA-seq workflows often depend on dedicated workflow managers, substantial computational infrastructure, or advanced bioinformatics expertise, which may limit their accessibility for routine analyses. We present a modular Bash- and Python-based batch-processing pipeline for estimating gene and isoform abundance and generating expression matrices from multiple RNA-seq runs. Using an SRA RunTable, a reference genome, and its corresponding gene annotation, the workflow automates reference preparation, sequencing-data retrieval, quality assessment, read preprocessing, alignment, abundance estimation, and post-processing. It produces gene-level TPM and FPKM matrices, individual gene- and isoform-level RSEM outputs, quality-control summaries, and integrated MultiQC reports. Configurable computational resources, sample-level status tracking, automatic download retries, selective reprocessing of failed samples, and controlled removal of intermediate files allow interrupted analyses to resume without repeating completed runs while reducing storage requirements. By combining batch processing, transparent configuration, and restartable execution in a lightweight workflow, this protocol provides an accessible approach for standardized RNA-seq abundance estimation.

Key features

• Processes multiple RNA-seq runs in batch, from sequencing-data retrieval and preprocessing to gene- and isoform-level abundance estimation.

• Supports paired-end and single-end libraries with configurable references, preprocessing parameters, and computational resources.

• Enables restartable execution with sample-level tracking, automatic download retries, selective reprocessing, and configurable cleanup of intermediate files.

• Generates gene-level TPM and FPKM matrices, gene- and isoform-level RSEM outputs, quality-control summaries, and aggregated MultiQC reports.

## Graphical overview


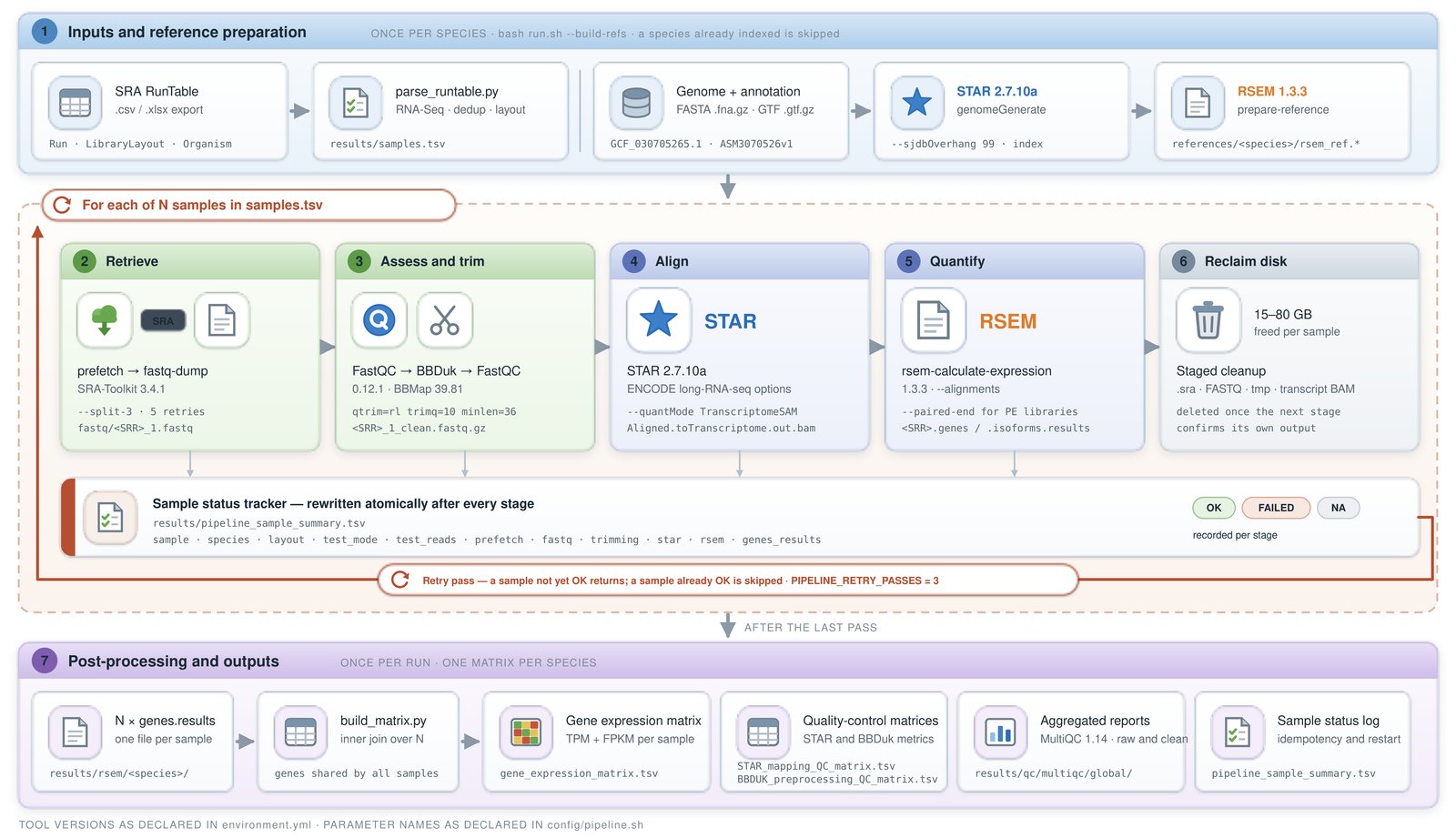


**Reproducible workflow for standardized processing of public RNA-seq datasets.** The pipeline automates data retrieval, preprocessing, expression quantification, quality-control reporting, and result generation from public sequencing data.

## Background

Public RNA-seq repositories provide access to extensive datasets generated across species, tissues, developmental stages, and experimental conditions. The NCBI Sequence Read Archive (SRA) enables the reuse of raw sequencing data for transcriptomic analyses beyond the objectives of the original studies [1]. These resources facilitate comparative analyses, hypothesis generation, and data integration without requiring additional sequencing experiments [2]. However, datasets generated by independent studies often differ in sequencing platform, read length, library layout, sequencing depth, metadata quality, and analytical processing, hindering their standardized integration and comparative interpretation. Several computational strategies are available for RNA-seq processing. Alignment-based workflows commonly use splice-aware aligners such as STAR [3] or HISAT2 [4], whereas lightweight approaches rely on pseudoalignment or selective-alignment tools such as kallisto [5] and Salmon [6]. In this pipeline, STAR is a requirement of the architecture rather than an interchangeable preference, because RSEM quantifies directly from the transcriptome-coordinate BAM file that STAR produces via --quantMode TranscriptomeSAM; existing protocols guide users through quality assessment, read preprocessing, alignment, transcript abundance estimation, differential expression analysis, and downstream visualization [7,8]. More comprehensive solutions based on workflow managers, including Nextflow and nf-core/rnaseq, provide scalable and portable processing across computing environments [9,10]. Nevertheless, these approaches may require familiarity with workflow-management systems, substantial computational infrastructure, or advanced bioinformatics expertise. Furthermore, the selection and version of alignment and quantification tools can influence abundance estimates and downstream analyses, particularly for low-abundance genes and transcripts with complex isoform structures [11,12]. This protocol presents a modular batch-processing pipeline for RNA-seq abundance estimation from multiple sequencing runs. The workflow integrates data retrieval, quality assessment, read preprocessing, alignment, transcript abundance estimation, and matrix generation within a transparent and configurable framework. Gene- and isoform-level abundances are estimated using RSEM, which accounts for ambiguously mapped reads and has demonstrated competitive performance relative to alternative RNA-seq quantification methods [11,13]. The pipeline generates standardized TPM and FPKM gene abundance matrices together with individual RSEM abundance files, quality-control summaries, and aggregated MultiQC reports. Transcript-level estimates, reported as individual isoform-level (.isoforms.results) outputs, are deliberately preserved alongside the gene-level files to support downstream gene-level inference through tools such as tximport combined with DESeq2 or edgeR, following Soneson et al. [14], when differential expression analysis is the end goal. This pipeline is executed directly on Linux workstations or servers and does not require a dedicated workflow manager. It incorporates sample-level status tracking, automatic download retries, restartable execution, configurable computational resources, and controlled removal of intermediate files. These features enable reproducible and storage-efficient batch processing of multiple RNA-seq runs while avoiding unnecessary repetition of successfully completed analyses. The workflow can be applied to any organism for which compatible reference genomes, gene annotations, and RNA-seq metadata are available.

## Equipment

1. Linux workstation or server with a multicore processor and Bash support

2. Random-access memory (RAM), at least 32 GB recommended

3. Local or network storage with sufficient capacity for SRA, FASTQ, BAM, reference, and result files

4. Stable broadband internet connection for downloading sequencing data, reference files, and software dependencies

*Note: The workflow is hardware-independent and can be executed on different workstation or server configurations. Therefore, manufacturer and model information are not applicable.*

## Software and datasets

**Software**

1. SRA Toolkit v3.4.1 [15]; public domain; released 2026-04-19

2. FastQC v0.12.1 [16]; GPL ≥3; released 2023-03-04

3. MultiQC v1.14 [17]; GPLv3; released 2023-01-08 (requires setuptools <81, pinned in environment.yml)

4. BBMap/BBDuk v39.81 [18]; BSD-3-Clause-LBNL; released 2026-03-31

5. STAR v2.7.10a [3]; GPLv3; released 2022-01-15

6. RSEM v1.3.3 [13]; GPL-3.0-or-later; released 2025-10-14 (the tool's own --version banner reports v1.3.1)

7. Python v3.11.16 [19]; Python-2.0; released 2026-09-02 (exact pin; not "or later")

8. openpyxl v3.1.5 [20]; MIT; released 2026-09-04 (exact pin)

9. GNU Wget v1.25.0 [21]; GPL-3.0-or-later; released 2026-02-27 (exact version used in validation)

10. Conda v26.5.3 [22]; BSD-3-Clause; released 2026-06-16

11. Git ≥ 2.30 [23] (used to clone the repository; not Conda-managed)

12. Bash ≥ 4.4 [24] (uses mapfile, printf -v, and associative arrays)

13. curl or GNU Wget [25] (either satisfies reference/RunTable downloads)

14. flock (util-linux) [26], optional (enables the run lock; without it, run.sh starts unlocked)

*Note: All Conda-managed versions above are exact pins in environment.yml (not minimum or "or later" versions). Release dates are those of the exact package build installed from its channel, which is the date that matters for reproduction. A frozen, fully explicit package list (environment.lock.txt, generated with conda list --explicit) is archived with the Zenodo DOI for exact reproduction of the validated environment.*

**Required input files**

• SRA RunTable (CSV or XLSX)

• Reference genome (FASTA)

• Gene annotation (GTF)

*Note: This workflow supports standard paired-end and single-end RNA-seq libraries, extracted with --split-3 during FASTQ conversion. Mate-pair libraries are a long-insert genomic preparation rather than an RNA-seq library type and are therefore out of scope. Processing mate-pair data would require different STAR alignment settings, including mate orientation and --alignMatesGapMax, and is not supported by the current pipeline version.*

## Procedure

This protocol processes RNA-seq datasets from SRA retrieval to gene- and isoform-level abundance estimation. The workflow includes computational environment setup, pipeline configuration, reference preparation, metadata parsing, read preprocessing, STAR alignment, RSEM quantification, quality-control reporting, and expression-matrix construction. Run all commands from the project root directory. The complete workflow is available from GitHub at https://github.com/lordziegler/OmniQuant-seq, and the version described here is permanently archived in Zenodo under DOI: 10.5281/zenodo.22750320 (both accessed September 14, 2026).

**A. Before you begin**

Before creating the pipeline environment, verify that Conda is installed.

conda --version

Once the environment has been created and activated in step A2, confirm that all required software is correctly installed by running:

echo "Verifying Conda environment and required software..." && \

conda --version && \

prefetch --version && \

fastqc --version && \

multiqc --version && \

STAR --version && \

rsem-calculate-expression --version && \

python --version && \

echo "All required software was detected successfully."

**Expected result:** The version of each program is displayed without error messages. If any command fails, verify that the Conda environment is active and that all dependencies were installed successfully before continuing.

If multiqc --version fails with ModuleNotFoundError: No module named 'pkg_resources', the environment has a setuptools newer than 81, which removed that module. Reinstall it pinned: conda install "setuptools<81".

1. Clone the pipeline repository and enter the project directory:

git clone https://github.com/lordziegler/OmniQuant-seq.git

cd OmniQuant-seq

2. Create and activate the Conda environment using the provided environment.yml file:

conda env create -f environment.yml && conda activate omniquant-seq

**Critical:** Do not proceed if one or more required programs are unavailable. Recreating or updating the Conda environment is recommended.

3. Run the included test suite to verify the basic pipeline functions:

bash tests/test_pipeline.sh

*Note: The test suite evaluates metadata parsing, input detection, file validation, library-layout normalization, species and analysis configuration, the command-line interfaces, and failure-cleanup behavior. It also covers the RunTable safety gates (--allow-genomic-source and --assume-layout), the read-length and index-overhang warning, reference checksum verification, the STAR strandedness column, and the inner join used to build the expression matrix. It does not execute SRA retrieval, read alignment, or expression quantification.*

**Expected result:** The command should finish with a summary similar to:

Results: 101 passed, 0 failed.

**B. Pipeline configuration**

1. Run the interactive configuration script. Both entry points open a menu when called without arguments and list every available mode with --help:

bash setup.sh

2. Specify the number of threads allocated to downloading, FastQC, BBDuk, STAR, and RSEM, and define the maximum memory available for STAR, the maximum allowed SRA download size, and the free-disk-space warning threshold. These values are written to config/pipeline.sh by the script.

3. Select the species to be processed. The setup script displays the species defined in config/species.sh and allows individual entries to be activated, deactivated, added, or removed.

The analytical parameters are set from a second menu of the same script, which writes them to config/pipeline.sh:

bash setup.sh --analysis

The parameters offered are each validated against their accepted range or list (Table 1).

**Table 1.** Configurable analytical and preprocessing parameters used by the RNA-seq pipeline

Parameter	Purpose	Default	Accepted range or type	Change it when
TEST_MODE	Restricts every sample to TEST_READS reads	false	true/false	Dry-running a new RunTable or reference before a full run
TEST_READS	Reads processed per sample in test mode	100000	positive integer	Faster or slower smoke tests are required
PIPELINE_RETRY_PASSES	Passes over samples.tsv retrying failed samples	3	positive integer	Flaky network conditions or SRA mirrors require more passes; a stable connection requires fewer
PREFETCH_RETRIES	Retries for a single prefetch download	5	positive integer	The connection is unstable
PREFETCH_RETRY_SLEEP	Seconds between prefetch retries	30	positive integer (seconds)	Rate-limited mirrors require a longer backoff
STAR_OVERHANG	STAR sjdbOverhang at index-build time	99 (assumes 100-nt reads)	non-negative integer, ideally max_read_length – 1	Datasets have a different typical read length. One index serves every run of a species, so mixed read lengths require a judgment call; the parser warns when a run's AvgSpotLen departs substantially from this value
STAR_SA_INDEX_NBASES	STAR genomeSAindexNbases	12	integer; STAR's guidance is min(14, log2(GenomeLength)/2 – 1)	Small genomes (< ~1 Gb) are processed: too high a value wastes RAM, too low a value reduces alignment speed
BBDUK_QTRIM	Read ends quality-trimmed by BBDuk	rl (both ends)	rl/r/l/f (BBDuk syntax)	Only one end shows quality problems
BBDUK_TRIMQ	Quality-trimming threshold (Phred)	10	0–40	Raw-read FastQC reports indicate that stricter or looser trimming is warranted
BBDUK_MINLEN	Minimum read length retained after trimming	36	positive integer (bp)	Library reads are shorter or longer, or a downstream minimum applies
BBDUK_REF	Adapter FASTA file used for adapter clipping	empty (disabled)	path to a FASTA file, or empty	Raw-read FastQC reports show adapter contamination

Command-line safety flags of helpers/parse_runtable.py, which are not written to config/pipeline.sh, are shown in Table 2.

**Table 2.** Command-line safety flags used during SRA RunTable parsing and sample preparation

Flag	Purpose	Default	Change it when
--allow-genomic-source	Also accepts rows whose LibrarySource is GENOMIC	off (TRANSCRIPTOMIC only)	DNA-seq-sourced runs are deliberately required for a specific analysis
--assume-layout PAIRED|SINGLE	Assigns a layout to rows whose LibraryLayout cannot be determined	unset (such rows are excluded)	The true layout of the excluded runs has been confirmed in the original metadata
--star-overhang N	Warns when a run's AvgSpotLen departs substantially from N+1	unset (no warning)	Always in practice: steps/parse_samples.sh passes STAR_OVERHANG automatically

*Notes:*

*1. The default value of STAR_OVERHANG=99 assumes 100-nt reads. One index serves every run of a species, so this parameter is set from the typical read length of the batch; the RunTable parser warns when the AvgSpotLen of a run differs substantially from STAR_OVERHANG + 1.*

*2. Adapter removal is disabled when BBDUK_REF is empty. In that configuration, BBDuk performs quality trimming and minimum-length filtering only. Whether adapter removal is necessary should not be assumed: inspect the raw-read FastQC reports in fastqc_out/ before deciding. Adapter content flagged in the Overrepresented sequences or Adapter Content modules indicates that BBDUK_REF should be set to an adapter FASTA file, whereas a clean adapter-content plot indicates that the default quality and length trimming (BBDUK_QTRIM, BBDUK_TRIMQ, and BBDUK_MINLEN) is sufficient on its own.*

**Critical:** Use a genome assembly and GTF annotation from the same reference release.

4. Prepare the reference genome and annotation

In the current pipeline version, process one species per working directory and activate only that species. Species entries are added, activated, deactivated, and removed from the species menu, which writes config/species.sh:

bash setup.sh --species

5. Prepare the input files

Before running the pipeline, place the SRA RunTable in the project root directory. Reference files are downloaded automatically from the URLs registered in config/species.sh, so supplying them manually is optional. The workflow automatically detects:

• One compressed reference genome in FASTA format (*.fna.gz, *.fa.gz, or *.fasta.gz).

• One compressed gene annotation in GTF format (*.gtf.gz).

• One SRA RunTable in CSV or XLSX format (*.csv or *.xlsx).

To supply the reference files manually instead of letting the pipeline download them, obtain the genome assembly and its corresponding annotation from the same NCBI reference release. For the example analysis using *Helicoverpa armigera*:

wget https://ftp.ncbi.nlm.nih.gov/genomes/all/GCF/030/705/265/GCF_030705265.1_ASM3070526v1/GCF_030705265.1_ASM3070526v1_genomic.gtf.gz

wget https://ftp.ncbi.nlm.nih.gov/genomes/all/GCF/030/705/265/GCF_030705265.1_ASM3070526v1/GCF_030705265.1_ASM3070526v1_genomic.fna.gz

Both reference files correspond to assembly GCF_030705265.1 (ASM3070526v1), released 2023-08-14, with RefSeq annotation release RS_2024_03 (March 2024). Both NCBI FTP targets were accessed on September 12, 2026.

For this example, the project root directory should contain:

GCF_030705265.1_ASM3070526v1_genomic.fna.gz

GCF_030705265.1_ASM3070526v1_genomic.gtf.gz

SraRunTable.csv

Export the sequencing metadata from the NCBI SRA Run Selector as a CSV or Excel (XLSX) file. The RunTable must retain the original header provided by NCBI and contain one row for each sequencing run.

For example:

Run,AssayType,AvgSpotLen,Bases,BioProject,BioSample,Bytes,...

SRR1015459,RNA-Seq,529,46818238,PRJNA218839,SAMN02378785,...

SRR1588177,RNA-Seq,72,618179303,PRJNA258614,SAMN02400131,...

DRR872486,RNA-Seq,302,6306747540,PRJDB39551,SAMD01751366,...

SRR10873990,RNA-Seq,300,7061752800,PRJNA600707,SAMN13830013,...

The pipeline automatically extracts the required metadata (Run, Assay Type, LibrarySource, LibraryLayout, and Organism) from the RunTable, accepting for each field any of the documented column-name aliases. Therefore, do not remove these columns or rename them to headings outside the accepted aliases after downloading the file from NCBI.

**Critical:** Keep only one SRA RunTable in the project root directory. When reference files are supplied manually, keep exactly one genome FASTA and one GTF annotation and activate a single species; with several species active, the local files are ignored, and each reference is downloaded from its own URL.

*Note: The SraRunTable.csv file is not downloaded automatically by the commands above. It must be exported from the SRA Run Selector after selecting the runs to be included in the analysis.*

6. Build the STAR index and RSEM reference:

bash run.sh --build-refs

7. For each active species, confirm that the following directories and files were created:

ls references/Helicoverpa_armigera/genome.fa

ls references/Helicoverpa_armigera/STAR_genome_index

ls logs/Helicoverpa_armigera_star_index.log

*Note: Reference preparation is restartable. Species with an existing STAR index and RSEM reference are skipped.*

**Pause point:** The workflow can be stopped after reference preparation. The reference files generated can be reused in later runs.

8. Prepare and inspect the sample list

The pipeline automatically parses the SRA RunTable when the workflow starts. To generate the sample list separately for inspection or troubleshooting, run the following command from the project root directory:

python3 helpers/parse_runtable.py \

 --input SraRunTable.csv \

 --output results/samples.tsv

**Expected result:** The parser reports the number of records loaded, the number retained after filtering for RNA-seq data, and the number of samples written to:

ls results/samples.tsv

The parser performs the following operations:

a. Retains records whose Assay Type or AssayType field is RNA-Seq.

b. Retains records whose LibrarySource field is TRANSCRIPTOMIC. Records declared GENOMIC are rejected unless --allow-genomic-source is passed explicitly.

c. Derives a species key from the Organism field using the format Genus_species, for example:

*Helicoverpa armigera* → Helicoverpa_armigera

d. Retains valid sequencing-run accessions beginning with SRR, ERR, or DRR.

e. Removes duplicate run accessions.

f. Normalizes the library layout to PAIRED or SINGLE. Records whose layout cannot be determined are excluded rather than assumed to be paired-end, unless --assume-layout PAIRED or --assume-layout SINGLE is passed explicitly.

The minimum required columns and their accepted aliases are listed below; the parser accepts any one alias per field:

Run accession — "Run" | "Run Accession" | "RunAccession" | "Accession"

Must match ^[SED]RR[0-9]+$ (SRR, ERR, or DRR).

Assay type — "Assay Type" | "AssayType" | "assay_type"

Must equal "RNA-Seq" for the record to be considered.

Library source — "LibrarySource" | "Library Source" | "library_source"

TRANSCRIPTOMIC by default. GENOMIC is rejected unless --allow-genomic-source is passed explicitly.

Library layout — "LibraryLayout" | "Library Layout" | "library_layout"

One of PAIRED, PAIRED END, or PE, or one of SINGLE, SINGLE END, or SE (case-insensitive). A record matching none of these is excluded rather than assumed to be paired-end, unless --assume-layout PAIRED or --assume-layout SINGLE is passed.

Organism — "Organism" | "organism" | "scientific_name"

Free text of the form "Genus species"; the first two whitespace-separated tokens form the species key (Genus_species). Empty or unresolvable values fall back to --fallback when it is given, and the record is otherwise dropped.

A field with no matching alias is treated as empty for that record. A ready-to-use minimal RunTable is distributed with the repository as examples/SraRunTable.example.csv.

Open and inspect the generated sample list:

head results/samples.tsv

The file contains three tab-delimited columns:

SRR SPECIES LAYOUT

SRR1015459 Helicoverpa_armigera SINGLE

SRR1588177 Helicoverpa_armigera SINGLE

DRR872486 Helicoverpa_armigera PAIRED

SRR10873990 Helicoverpa_armigera PAIRED

Verify that:

• All intended runs are present.

• The species key matches the reference configured in config/species.sh.

• Each library layout is correctly assigned.

• No unwanted or duplicate runs remain.

**Critical:** Do not pass --assume-layout without first confirming the true layout of the excluded runs in the original SRA metadata. An incorrect layout may prevent FASTQ extraction or cause the wrong input files to be used.

**Pause point:** Review and, if necessary, edit results/samples.tsv before downloading sequencing data. Removing unsuitable runs or correcting species and library-layout assignments at this stage avoids downloading and processing data that are subsequently discarded.

9. Run a reduced test analysis

Execute the workflow in test mode:

bash run.sh --test

*Note: In test mode, the pipeline processes only the number of reads specified by TEST_READS, which is 100,000 reads per sample by default.*

10. Confirm that the test run produces:

results/rsem/<species>/<SRR>.genes.results

results/rsem/<species>/<SRR>.isoforms.results

results/pipeline_sample_summary.tsv

results/tables/

results/qc/

11. Review the test-run logs and outputs before proceeding to the complete analysis.

**Critical:** Do not combine test-mode and full-run results in the same final expression matrix. Remove the test results or use a separate working directory before initiating production processing.

**C. Execute the complete RNA-seq processing workflow**

Start the complete analysis:

bash run.sh --full

For every sample listed in samples.tsv, the pipeline performs the following operations.

1. Retrieve the SRA run

The workflow downloads the run with prefetch. Failed downloads are retried according to PREFETCH_RETRIES and PREFETCH_RETRY_SLEEP.

2. Convert the SRA run to FASTQ

*Paired-end runs generate:*

<SRR>_1.fastq

<SRR>_2.fastq

*Single-end runs generate:*

<SRR>.fastq

3. Assess raw-read quality

The workflow runs FastQC on the raw FASTQ files and stores the reports under fastqc_out/.

4. Perform read trimming and filtering

BBDuk performs quality trimming and minimum-length filtering using the parameters specified in config/pipeline.sh.

For paired-end libraries, the main outputs are:

clean_fastq/<SRR>_1_clean.fastq.gz

clean_fastq/<SRR>_2_clean.fastq.gz

clean_fastq/<SRR>_singletons.fastq.gz

The singletons file contains reads whose mate was removed during trimming.

For single-end libraries, the output is:

clean_fastq/<SRR>_clean.fastq.gz

When an adapter reference is supplied through BBDUK_REF, BBDuk also performs adapter trimming.

5. Assess trimmed-read quality

FastQC is run again on the cleaned reads. MultiQC then generates a sample-level report containing the available quality-control results.

6. Align the cleaned reads with STAR

The pipeline aligns the reads against the species-specific STAR index and requests two outputs through --quantMode TranscriptomeSAM GeneCounts: the transcriptome-coordinate BAM file required by RSEM, and the per-gene strand-resolved count table (ReadsPerGene.out.tab) from which the library strandedness of each sample is inferred before quantification.

The effective invocation is:

STAR --runThreadN <THREADS_STAR> --genomeDir <STAR index> \

 --readFilesCommand cat|zcat --outFileNamePrefix <prefix> \

 --readFilesIn <reads> \

 --outSAMtype BAM Unsorted --outSAMunmapped Within \

 --outFilterType BySJout --outSAMattributes NH HI AS NM MD \

 --outFilterMultimapNmax 20 --outFilterMismatchNmax 999 \

 --outFilterMismatchNoverReadLmax 0.04 \

 --alignIntronMin 20 --alignIntronMax 1000000 --alignMatesGapMax 1000000 \

 --alignSJoverhangMin 8 --alignSJDBoverhangMin 1 --sjdbScore 1 \

 --quantMode TranscriptomeSAM GeneCounts

These are the ENCODE long-RNA-seq settings and are identical for every organism. The organism-dependent values, STAR_OVERHANG and STAR_SA_INDEX_NBASES, are applied when the index is built rather than at this step.

The final STAR log and the per-gene count table are copied to:

logs/<SRR>_STAR_Log.final.out

logs/<SRR>_STAR_ReadsPerGene.out.tab

**Critical:** RSEM requires the transcriptome BAM generated by --quantMode TranscriptomeSAM. The sample is marked as failed if this file is absent.

7. Quantify gene and isoform abundance with RSEM

The --paired-end option is included only for paired-end libraries.

The effective invocation is:

rsem-calculate-expression --alignments --num-threads <THREADS_RSEM> \

 --temporary-folder <tmp> --forward-prob <inferred> [--paired-end] \

 <transcriptome BAM> <RSEM reference> <output prefix>

The --forward-prob option is set per sample rather than left at the RSEM default. STAR is run with --quantMode TranscriptomeSAM GeneCounts, which requires no additional pass because STAR already parses the annotation, and writes ReadsPerGene.out.tab. The forward and reverse columns of that file are summed into the ratio r = fwd/(fwd + rev): r > 0.8 sets --forward-prob 1, r < 0.2 sets --forward-prob 0, and intermediate values set 0.5, corresponding to an unstranded library. This ratio is also reported per sample in STAR_mapping_QC_matrix.tsv. Quantifying every library as unstranded regardless of its actual protocol misassigns reads between genes that overlap on opposite strands, a relevant risk when SRA runs from different laboratories are aggregated.

The principal outputs are:

<SRR>.genes.results

<SRR>.isoforms.results

These files contain expected counts, TPM, FPKM, effective lengths, and related abundance information.

8. Record the sample status and remove intermediate files

After each stage, the pipeline updates:

results/pipeline_sample_summary.tsv

The file records:

sample

species

layout

test_mode

test_reads

prefetch_status

fastq_status

trimming_status

star_status

rsem_status

genes_results

Completed samples are skipped when the same command is run again. Failed samples are retried during subsequent passes through the sample list.

Depending on the cleanup options in config/pipeline.sh, the workflow may remove downloaded SRA files, raw FASTQ files, cleaned FASTQ files, temporary STAR files, temporary RSEM files, and intermediate BAM files after successful completion.

*Note: Review the cleanup settings before execution when intermediate files are required for troubleshooting or independent reanalysis.*

**D. Generate expression and quality-control matrices**

1. Post-processing runs automatically after the sample-processing loop. To execute it separately, use:

python3 helpers/build_matrix.py \

 --rsem-dir results/rsem \

 --output results/tables/gene_expression_matrix.tsv \

 --star-logs logs \

 --bbduk-logs logs \

 --star-out results/tables/STAR_mapping_QC_matrix.tsv \

 --bbduk-out results/tables/BBDUK_preprocessing_QC_matrix.tsv

2. Confirm that the following tables were generated:

results/tables/gene_expression_matrix.tsv

results/tables/STAR_mapping_QC_matrix.tsv

results/tables/BBDUK_preprocessing_QC_matrix.tsv

The expression table contains the gene identifier, its transcript identifiers, length, effective length, and expected count, followed by one TPM column and one FPKM column per sample. Genes are matched across samples by gene_id, and only genes present in every sample's .genes.results file are retained in the matrix.

The schema of each table is as follows:

gene_expression_matrix.tsv (tab-delimited)

gene_id (text), transcript_id(s) (text, comma-separated), length (integer), effective_length (decimal), and expected_count (decimal), followed by two columns per sample: <sample>_TPM (decimal) and <sample>_FPKM (decimal). The gene_id, length, effective_length, and expected_count values are taken from the first RSEM file in which each gene appears and are therefore not the values of any single sample. Use each sample's own .genes.results file when sample-level counts or effective lengths are required.

STAR_mapping_QC_matrix.tsv (tab-delimited)

STAR_metric (text), one row per line of the STAR Log.final.out file, for example “Uniquely mapped reads %” or “% of reads mapped to multiple loci”, followed by one column per sample. The table includes a strand_ratio row, fwd/(fwd + rev), in which a value near 0.5 indicates an unstranded library and a value near 0 or 1 a strand-specific one.

BBDUK_preprocessing_QC_matrix.tsv (tab-delimited)

Sample (text), followed by Input, QTrimmed, Total_Removed, and Result, each reported as _reads (integer), _reads_percent (decimal), _bases (integer), and _bases_percent (decimal), parsed from the BBDuk summary lines. Input has no percentage columns because it is the 100% baseline; those cells are NA by design rather than missing data.

*** Representative rows with real values for the three tables above.

3. Confirm that the number of samples in the expression matrix agrees with the number of samples marked rsem_status=OK in pipeline_sample_summary.tsv.

4. For the current pipeline release, construct and interpret expression matrices separately for each species.

**Critical:** Do not merge gene identifiers from different species unless an explicit orthology table and a dedicated cross-species aggregation procedure are used.

*Note: The expected_count and effective_length values in individual RSEM files are sample-specific.*

**E. Review the final outputs**

1. Inspect pipeline_sample_summary.tsv and confirm that all retained samples have an OK status for the required stages.

2. Review the raw- and cleaned-read MultiQC reports and the STAR and BBDuk quality-control reports.

3. Exclude unsuitable samples before downstream biological interpretation. The thresholds recommended below for flagging a sample are conventional values for STAR and BBDuk metrics and are intended as a starting point, to be compared against the distribution of the whole batch rather than applied in isolation (Table 3).

**Table 3.** Recommended quality-control metrics and decision thresholds for evaluating RNA-seq samples.

Metric	Source	Threshold	Action
Uniquely mapped reads %	STAR	below 60%	Inspect the sample; commonly, a degraded library or a mismatched reference
% of reads mapped to multiple loci	STAR	above 30%	Inspect for repetitive-element or rRNA contamination
% of reads unmapped: too short	STAR	above 10%–15%	Adapters were probably not fully trimmed; revisit BBDUK_REF
strand_ratio, fwd/(fwd + rev)	STAR	out of line with the rest of the batch	Check the actual library preparation of the run; do not pool mixed-protocol batches without accounting for the difference
Result_reads as % of Input_reads	BBDuk	below 70%	Trimming was aggressive; inspect the raw-read FastQC report before accepting the sample

Exclude a sample that fails two or more of these criteria with no plausible biological explanation. Reprocess a sample that fails a single metric for a reason that a configuration change addresses, such as supplying an adapter reference. Retain, with the reason documented, a sample that is marginal on one metric but whose depth and coverage remain adequate for the intended analysis.

4. Preserve the following files with the final analysis:

environment.yml

environment.lock.txt (conda list --explicit output)

config/pipeline.sh

config/species.sh

samples.tsv

pipeline_sample_summary.tsv

gene_expression_matrix.tsv

STAR_mapping_QC_matrix.tsv

BBDUK_preprocessing_QC_matrix.tsv

MultiQC reports

individual RSEM result files

## Validation of protocol

This protocol is used in:

• Pejendino et al. [2]. RNA-seq Co-Expression Analysis Reveals a Midgut-Associated Digestive Gene Module in *Helicoverpa armigera. BioTech*, 15(3), 53. https://doi.org/10.3390/biotech15030053.

**Expected outputs and downstream applications**

Successful completion of the workflow produces the quality-control files described above and a gene-level expression matrix in results/tables/gene_expression_matrix.tsv. For downstream expression analyses, the principal analytical output is the TPM component of this matrix. Each row represents a gene identifier, whereas each sample column corresponds to one SRA run included in the input RunTable. Consequently, the number and identity of sample columns depend directly on the list of SRA accessions provided by the user. Processing a different set of SRA runs, therefore, produces an equivalent gene-by-sample TPM matrix containing the corresponding samples.

A representative excerpt of the TPM matrix generated by the workflow is shown in Table 4. In this example, the columns correspond to the SRA accessions ERR973500–ERR973506, and the rows correspond to individual gene identifiers. The complete output contains the TPM abundance estimates for all genes retained after processing the complete set of supplied RNA-seq runs.

**Table 4.** Representative excerpt of the gene-by-sample TPM matrix generated by the workflow.

Gene	ERR973500	ERR973501	ERR973502	ERR973503	ERR973504	ERR973505	ERR973506
LOC110371794	0.11	0.05	0	0.15	0.23	0.16	0.10
LOC110372098	0.25	0.06	0	0.24	0.08	0	0
LOC110372412	42.92	27.46	49.32	13.41	24.09	7.32	26.53
LOC110372637	15.43	8.12	9.18	10.17	9.71	6.46	3.57
LOC110376134	0.41	0.31	9.46	0.17	0	0	0
LOC110376142	0.16	0	0.11	0	0	0	0
LOC110376161	0	0	0	0	0	0	0
LOC110376168	0	0	0.20	0.37	0	0.40	0.50
LOC110378553	0	0.08	0.11	0	0.23	0	0.29
LOC110378558	0	0.07	0.21	0	0.11	0.11	0
LOC110378564	0.30	0	0.10	0.10	0.20	0.10	0
LOC110378565	674.17	408.50	444.11	542.32	702.35	608.63	388.98

The resulting TPM matrix constitutes the principal expression-level endpoint of the present protocol and can subsequently be used as input for downstream transcriptomic analyses. Depending on the biological question, these analyses may include principal component analysis (PCA), sample-to-sample correlation and clustering, expression profiling, or weighted gene co-expression network analysis (WGCNA), after applying the filtering and transformation procedures required by each method. These downstream analyses are intentionally outside the scope of the present protocol. Thus, the workflow described here ends with the generation and quality assessment of the standardized gene-abundance matrix, while individual RSEM gene- and isoform-level files and quality-control reports are retained to provide traceability and facilitate further analyses.

The utility of this type of expression matrix for downstream biological analysis was demonstrated in the previously published application of the workflow by Pejendino et al. [2]. That study compiled 579 publicly available *Helicoverpa armigera* RNA-seq libraries from 54 independent experiments, from which a harmonized subset of 130 libraries was subsequently used for WGCNA. The resulting expression data supported the module–trait correlation analysis presented in Figure 1 (from [2]), the comparison of module eigengene distributions shown in Figure 2 (from [2]), and the TPM-based expression profiles of serine protease genes presented in Figure 3 (from [2]). These analyses illustrate how the standardized abundance matrix produced by the RNA-seq processing workflow can serve as the starting point for subsequent biological and network-level analyses.
